# Six Cases of Phthisis Pulmonalis Treated by Cod-Liver Oil

**Published:** 1851-04

**Authors:** David W. Yandell

**Affiliations:** Louisville, Ky. Formerly attending Physician for the Faculty, at the Louisville Marine Hospital: One of the physicians to that Institution, etc.


### Art. II.—*Six cases of Phthisis Pulmonalis treated by Cod-Liver Oil.*

By David W. Yandell, M. D., of Lonisville, Ky. Formerly attending Physician for the Faculty, at the Louisville Marine Hospital: One of the physicians to that Institution, etc.

Case I.—Was a married lady, aged 28 years, of imperfectly developed form and delicate habit, with freckled skin, red hair, and high nervo-lymphatic temperament, who inherited from her mother a deep tuberculous taint.

Her general health had never been very good. She had never borne children.

The patient does not remember ever to have been without a cough during the colder seasons of the year. It was sometimes violent and paroxysmal, but generally, short, frequent and hacking in its character. She has had two attacks of acute pleurisy, both affecting the left side, and, according to the testimony of her physicians, confined to the central and upper portions of the left lung, and of very limited extent. Towards the close of the spring of 1849, the patient had a slight hemoptysis, which was almost immediately followed by acute inflammation of the summit of the left lung, implicating both the pleura and pulmonary parenchyma. Her medical attendant pursued the treatment usual in such cases, which, however, produced no satisfactory change in the symptoms, and at the expiration of six weeks he was dismissed. During this time, it may not be unworthy of remark, the hemoptysis recurred on several occasions, once or twice to a considerable extent. I was called to the patient on the first of July, when her condition was briefly as follows: She was the subject of hectic and night sweats. Her respiration was hurried and painful; cough frequent and short; expectoration muco-purulent; pulse quick and irritable; bowels costive, appetite poor, thirst considerable. She complained of excessive pain of a lancinating character at the summit of the left lung, which frequently extended from this point over the whole of that side. It was increased by cough and full inspiration. The patient had emaciated very rapidly, and was exceedingly feeble—too much so to leave her room, and generally, to quit her bed.

*Exploration of the Chest.*—The chest was narrow and flattened antero-posteriorly, and dilated very imperfectly. *Right side*—reasonance normal; puerile respiration at the summit. *Left side.* Dulness in clavicular region and in corresponding portion of lung posteriorly. Respiratory murmur feeble over the same regions, mingled with a mucous ronchus, composed of fine and frequent bubbles; bronchial respiration and slight broncophony, more perceptible, perhaps, behind than in front, and a grazing friction sound.

In view of the local complication—pleurisy—I ordered a blister to be applied over its seat, to be dressed with a cerate containing morphine. Not deeming this a suitable time to commence the cod-liver oil, I met the different indications by anodyne expectorants, chalybeates, vegetable tonics and aromatic sulphuric acid, and, as I have already mentioned, counter irritation. The most prominent and distressing symptom—the pain, disappeared after the application of the second blister. Not so with the hectic, night sweats and cough. These persisted, although, perhaps, in diminished degree, for more than two weeks, notwithstanding the exhibition of all the remedies of repute for such symptoms. At the end of this time I determined to resort to the oil, and commenced by administering it in the following combination:

℞ Cod-liver oil ℥iv.

P. Gum Arabic,

White sugar, of each ʒij.

Peppermint water, ℥iv.—Mix.

Dose, two table spoonsful morning and evening, and taken about two hours after a meal. This quantity being well borne, and producing no sensible effect on the disease, I gradually increased it, and as tolerance was established, carefully substituted the pure oil of the mixture. Very soon, within four days, the appetite improved, the cough abated, the sputa diminished in quantity, the hectic and night sweats disappeared, and the pulse became slower and of better volume. The patient was now taking one ounce and a half and two ounces of the pure oil during the day. The physical signs at this time remained the same. The treatment was persisted in, the oil being gradually augmented to double the quantity, and within a fortnight the patient had improved perceptibly in both flesh and strength. Her cough, however, continued troublesome, and hoping to assist the action of the oil, I prescribed,

℞ Morph acet. gr. iij.

Tinct. sanguin. canad. ʒij,

Vin. ant. et potass. tartrat.

Vin. ipecac. aa. ʒiij.

Syrup. prunus virgin. ℥iij.—M.

Dose, from fifty to seventy drops several times a day. This I think, was of service.

It was now two months since the patient had taken the first dose of the oil, and I seldom, if ever, saw a more marked improvement in the general condition of any one in the same length of time. She had increased several pounds in flesh—her appetite was excellent, her pulse natural, skin pleasant. She slept soundly and was in high spirits. And what was equally gratifying, the mucous ronchus had almost entirely subsided, and the respiration had become clearer and drier. But the dulness, bronchial respiration and broncophony remained undiminished.

I now conjoined with the other treatment constant counter-irritation, alternately on the front and posterior aspects of the chest, and at the expiration of six weeks there was a slight increase in the resonance on percussion, and an improvement in the voice and breath sounds, though the latter continued distinctly tubular. The expectoration had ceased to be purulent and had become mucous, and was greatly diminished in quantity. Treatment continued.

In February, 1850, about seven months after I had taken charge of the patient, I was called to a neighboring village to which she had removed in the commencement of the winter, and while there made an attentive examination of her chest. There was still the slightest possible difference in the reasonance of the two sides, and a perceptible inequality in the breath and stroke sounds, with prolonged expiration which might, with strictness, be said to be even tubular in its character. "These signs," says Dr. Williams, "I have learned by the experience of many years, not to consider as exceptional against recovery, for they appear to be dependent on the puckering of the texture, often with pleural adhesions and old deposits in the bronchial glands, so frequently found after death at the summits and near the roots of the lungs of persons who have not for many years exhibited symptoms of any pectoral disease."

In point of general health, appearance, condition and so forth, the patient was never so well. She weighed more than she had ever done, and but for the cough which still persisted—though it would not have been observed by other than herself, and was followed by no expectoration whatever—she presented every external evidence of complete restoration to health. Throughout the summer and fall I continued to receive intelligence of her of the most gratifying character, invariably representing that but for the cough, which she now referred exclusively to the throat, she was in the enjoyment of the most perfect health. And although I have not heard from her now for several months, I have every reason to believe that her health continues uninterrupted.

Case II.—On the night of the 15th of Oct. 1849, J. Y., aged 22 years and a half, was attacked, without appreciable cause, with violent hemoptysis. My father, Professor Yandell, was called to see him, and by the administration of large quantities of opium and sugar of lead, succeeded in arresting the hemorrhage. The following morning I visited Mr. Y. in consultation, and found him suffering with the symptoms usual in hemoptysis when dependent on tubercles, to wit:—Face flushed and expressive of great anxiety; pulse full, frequent and hard; respiration accelerated; occasional cough followed by the expectoration of small quantities of florid and frothy blood.

There was nothing either in the descent, occupation, or physical conformation of Mr. Y. which predisposed, in any observable degree, to the lesion on which hemoptysis almost always depends, to-wit: tubercles. He was born of healthy parents, was a stout and apparently hearty man, a brick mason, of sober, industrious and temperate habits, with finely developed chest, who had never before had hemoptysis or cough. The most minute and careful exploration of the chest revealed no loss of integrity in the respiratory apparatus, unless it was a scarcely appreciable alteration in the rythm of the respiration, consisting in a slight prolongation of the expiratory sound. Resonance good; vesicular murmur soft, healthy; unattended by any of the rhonchi whatever.

It was believed that venesection was indicated and I accordingly bled Mr. Y. freely. The hemorrhage ceased; the pulse lowered, the sense of constriction about the chest of which he had complained, was removed, his breathing became natural, and his face lost the expression of anxiety which it had worn when I first entered the room. His bowels not having been opened for two days, a purgative was ordered, and with the view of preventing, as far as possible, the return of the hemorrhage, the patient was directed to take occasional doses of the sugar of lead, observe the most perfect quiet both of mind and body, use only the blandest and unirritating articles of food, and when thirsty, acidulated drinks.

I continued to see Mr. Y. daily, and sometimes twice a day, for several days, and at every visit I repeated the examination of his chest. Day by day the alteration in the respiratory murmur became more and more perceptible, and at the expiration of a week, there was evident dulness, and all the other positive signs of consolidation at the summit of the right lung. The change, it will be perceived, was rapid and of a character which required energetic treatment. Blood was abstracted immediately from the seat of the disease by the application of cups, which were followed, after being repeated two or three times, by the establishment of a large issue just beneath the clavicle which was kept open for several weeks. From the moment that the nature of the disease was clearly apparent the cod-liver oil was exhibited pure, and in large quantities. But notwithstanding, the tubercles, or, at least a portion of them, softened, and were expectorated in the characteristic form. Yet the original limits of the dulness did not increase, nor did the breath or voice sounds become manifestly more distinct, though the moist rhonchi were superadded to the auscultatory phenomena already mentioned.

The patient was in the finest general health; his appetite and digestion were excellent, and he increased considerably in flesh.

In the spring—April 1850—he had ceased to expectorate pus, and his cough, which was very rare, was followed only by the expectoration of small quantities of transparent mucus. Corresponding with this encouraging change, the moist rhonchi had entirely disappeared, and the tubular character of the respiration was notably less intense—which was also true of the voice and dulness—though still greatly too much so to warrant any but an extremely guarded prognosis.

The season for building having commenced, I advised the patient to resume his work, which he did, and continued until the cold weather in autumn. Throughout the whole of this time his general condition was most satisfactory. He was almost entirely free from cough, and did not expectorate. The signs of solidification were much less apparent than they were six months before, though still easily detected.

On the 17th inst., Mr. Y. called to say that he believed he was well. After a careful examination of his chest I am of the opinion that the diseased portion of lung has regained its clearness of sound on percussion, and but for having *expected* to find dulness believe I would have discovered no difference whatever in the two sides. One of my pupils, Mr. Kennedy, in whose knowledge of physical diagnosis I have much confidence, was unable to detect any difference in the resonance of the two sides—There was a slight inequality in the breath and stroke sounds, though less than was to be anticipated where there had been so short a time before such extensive disease: Voice sound healthy; expiratory sound still somewhat prolonged.

Mr. Y. weighs sixteen or seventeen pounds more than when he commenced the oil, and has become, he says, fond of it.

I regard this case as one in which, to say the least, the progress of the disease has been retarded. The lungs have, I think, unquestionably been brought back from the stage of incipient softening to that of simple deposit, "which," as has been observed by Dr. Williams, "is tardier in its changes of increase or diminution, and may remain long stationary without any obvious alteration."

CASE III. *History.*—Mrs. M. is a mulatto woman, aged 29 years; mother living and healthy; father died of hip disease; married; no children; health never very good; subject to colds; never had hemoptysis. During the winter of 1847–8, had a severe attack of what her physician called bronchitis; had been sick ever since; from that time had constant night sweats and hectic fever, a hard, frequent cough, copious purulent expectoration, dyspnea, pain in the left side; loss of appetite, occasional diarrhea.

*Condition at the time of visit in November* 1849.—Patient confined to bed, excessively feeble, emaciated, pale, anemic looking. Pulse small, quick; skin dry, cough frequent, painful; sputa abundant, purulent. Respiration hurried, difficult, short. Pain in the left side, in front and behind. Hectic in the afternoon followed by exhausting sweats. No appetite; thirst.

*Physical Signs.*—Left side dull in clavicular, sub-clavicular and mammary regions. Bronchial voice, cough and respiration, very fine mucous rhonchus in same localities. Signs of a small cavern immediately above the nipple. Tubal respiration posteriorly. Rude respiration over the right side.

*Treatment.*—Cod-liver oil. The improvement in the condition of the patient after having taken it a few days, was manifest—at the end of a few weeks it was marvelous. The night sweats and hectic had disappeared altogether, the cough had abated, the expectoration was less abundant and had become muco-purulent, the appetite had returned; the patient was able to be about her room; she had increased in flesh and strength, and but for great shortness of breath, would have been extremely comfortable. The only change which was at this time observable in the physical signs, consisted in a slight diminution in the frequency and regularity of the mucous rhonchus. The evidences of consolidation of the pulmonary parenchyma remained unaltered. In addition to the oil—which the patient was taking in doses of an ounce twice and three times a day—I effected counter irritation over the region of the dulness. This was kept up for several months, with, I think, the happiest results.

The patient kept her room throughout the winter. As soon as the weather became pleasant in the spring she commenced taking out-door exercise, and in the last week in April, walked to my office, distant a mile and a half from her house. The only inconvenience of which she complained afterwards was the shortness of breath. At that time—six months after I first saw her—the dulness had grown less, the mucous rhonchus had ceased, the voice, breath and cough sounds were less clear; the respiration on the right side had diminished in rudeness—the cough was only occasional, unattended by pain, and succeeded by the expectoration of small quantities of transparent mucus.

During the summer the patient continued to enjoy excellent general health, and but for the cough, would have indulged the hope that she was entirely restored. In September she was seized, after having been exposed to the night air, with pain in the left side, followed by high fever and aggravation of the cough. I was sent for but being absent from the city she was attended by another physician. The attack—which I take from her description of it to have been one of circumscribed pleurisy—confined her to bed for two weeks. Convalescence once fairly established, she soon regained her strength and flesh, but the cough continued hard, frequent and very distressing.

*Examination on the 20th Feb.* 1851.—General aspect and expression of patient good. Tissues solid, firm. Pulse and skin natural; appetite excellent. Rests well at night; attends to all the ordinary duties of house keeping. Breathing 20 in a minute, short. Cough hard, ringing; expectoration frothy, mucous, scanty. Dulness still perceptible, though the difference in the resonance of the two sides is very slight; respiration in the healthy lung puerile. Feebleness of the vesicular murmur over the whole of the left side. Respiration tubular in the mammary region. Prolonged expiatory sound; inequalities in the breath and stroke sounds. And yet, notwithstanding all this, *almost the entire left lung is permeable in some degree to the air.* Many of the cells have doubtless been entirely destroyed and others wholly obliterated; but others again, have sustained merely a loss of their normal elasticity. Auscultation over the middle and especially the inferior portions, both anteriorly and posteriorly, of the left side of the chest, conveyed the idea of a lung which had lost its elasticity, its natural capacity to expand and contract, rather than of a lung whose cells had been either destroyed or closed.

The only thing of which the patient complains is "shortness of breath." She is unable to walk rapidly or ascend stairs without a distressing sense of suffocation. The quantity of air capable of being contained in the chest is evidently much less than in health and by far too little for the purposes of physiological respiration. I regret that I am not provided with a spirometer by which I might ascertain the exact loss in the vital capacity of the respiratory apparatus.

Case IV.—Charles, a mulatto man, entered the Louisville Marine Hospital on the 29th August, 1849. *History. Aet.* 59 years, of healthy descent and a long lived family. Has been a very stout and able-bodied man. In July 1839, while in Lower Canada, had a slight hemorrhage from his lungs; has had several hemorrhages since. Health however, continued good until the autumn of 1844; since then had failed rapidly; did no work after that time; had pains in his left side, severe cough, emaciated, lost strength, had night sweats and hectic. Came to the Hospital, he said, to die.

*Condition at time of admission.*—Patient excessively emaciated and feeble. Pulse small, weak; skin dry; cough frequent, hard; expectoration abundant, purulent; respiration accelerated, performed principally by the right side. Anorexia, thirst, hectic every afternoon, exhausting night sweats.

*Physical Signs.*—Cracked pot sound, and other cavern signs, at the summit of the left lung, over a space about the size of a goose egg. Dulness below this to the infra mammary region. Mucous rhonchus; a little sibilous. Respiration tubal. Prolonged expiatory sound. Voice and cough ringing. Resonance of the right side normal. Respiration rude.

Ordered cod-liver oil and iron, counter irritation over the dulness. In a very few days the cough abated, the hectic and night sweats ceased, the appetite improved, thirst diminished and patient became more cheerful, said he was "a hundred per cent better." Slowly the ronchi disappeared, gradually the cells became permeable to the air, and the resonance clearer. Patient undertook the duties of nurse to the negro ward of the hospital, which place he continues to fill.

*Examination on Sunday, 23d inst.*—Patient has gained several pounds in weight; health good; coughs still, expectorates a frothy mucus; no hectic or night sweats; only complaint is shortness of breath, but for this and his cough, says he would be well; says he is incomparably better, in every respect, than he has been since the autumn of 1844; says he knows it is to the cod-liver oil that he owes his life.

*Physical Signs.*—Resonance of diseased lung greatly improved though still perceptibly less than on the healthy side. Cavern signs unchanged; no rhonchi; respiration feeble but rough and heard *over the whole of the limits of the dulness*, very distinct on full inspiration. Expiration more prolonged than I ever before observed in any case. Voice and cough sounds about as clear and ringing over the region of the solidification as they are in the first stage of pneumonia. Marked inequalities in the breath and stroke sounds. Respiration puerile on the right side.

Case V.—Mr. C. B...., a clerk, called to see me in July, 1850, to consult me relative to a cough, which he said was wearing him away.

*History.*—*Aet.* 25 years. Patient of small frame, florid complexion, blue eyes, light hair, married. Born of healthy parents, both of whom are now living at an advanced age; has several brothers and sisters, all healthy. When a child, had measles which left him with a cough—friends said he would outgrow it—had not done so. It was very troublesome, and often painful during cold weather; was a dry cough; had never alarmed him. Two years ago expectorated a small quantity of blood; had a recurrence of the hemorrhage in March last, became frightened, and endeavored to effect an insurance on his life. Was examined, he says, minutely by the physician to one of the Life Insurance Companies in this city, and was recommended as a person on whose life the Company would be safe in "taking a risk.' Unfortunately for Mr. B. the Board thought differently, and declined issuing a policy.

Soon after the last attack of hemoptysis he commenced losing flesh, appetite and strength; suffered with palpitations of the heart; was harassed by cough, which was no longer dry as it had previously been, but was followed by free expectoration of a ropy mucus, sometimes, he said, of the color of straw, and most abundant when he first rose in the morning. Took cold in the early part of July; cough was greatly aggravated, very painful; deprived him of sleep, said he thought sometimes it would kill him.

*Condition at time of examination in the last week in July* 1850.—Weighed 107 or 108 lbs. General appearance that of great feebleness. Complained of fulness at the top of right lung. Respiration accelerated, somewhat painful, short. Slight febrile excitement. Cough violent, occurring in frequent paroxysms. Expectoration muco-purulent. Chest narrrow, slightly flattened at apex; dilated imperfectly at summit of right lung.

*Physical Signs. Right Side.*—Dulness from the clavicle to the third rib inclusive. All the phenomena of consolidation of the lung at this point: in addition, an occasional mucous ronchus, very rare and composed of very large bubbles. Prolonged expiration: *Left Side*—Resonance good; respiratory murmur intensely puerile at the summit.

Patient complained several times during the examination of the sense of fulness at the superior portion of the right lung. I ordered cups over the part, and Mr. B.... afterwards told me that they removed the distress immediately. Patient was directed to take cod-liver oil in large quantities: says he commenced improving immediately, and for the first time in many months enjoyed uninterrupted rest at night. Shortness of breath grew less, palpitation disappeared, appetite and strength improved. His friends observed the wonderful improvement in his appearance. In spite, however, of this evident amelioration in Mr. B.'s general condition, the process of softening of the tubercles was not arrested, at least at the extreme summit of the lung, where a small cavern, about the size of a hen's egg, was formed. In other portions of the diseased lobe the march of the malady was retarded—perhaps altogether arrested. In connexion with the softening of the tubercular matter and formation of the cavern, I am pleased to record the same remarkable fact which was observed in Case No. 2, to-wit: *that throughout the whole process, which lasted during three months, the patient had neither feverish excitement nor night sweats.*

Without stopping to record the result of the frequent and repeated examinations which I made of Mr. B.'s chest throughout the summer and fall, I will close the report of his case by stating the condition of his lungs at this time, as revealed at an exploration made on the 24th instant. Before doing so, however, I deem the following circumstances worthy of remark. Mr. B. weighed, as I stated, in July last, 107 or 108 lbs. He now weighs between 124 and 125 lbs., an increase of seventeen pounds. His muscles are firm and solid; he rolls ten pins several hours every day without feeling fatigued; his complexion is healthful; he has a very slight and not at all troublesome cough; expectorates a small quantity of healthy mucus; says he is in perfect health, and has never before felt so stout and hearty.

*Physical Signs.*—Hardly perceptible dulness in regions where, in July, there was almost flatness. Cavern signs under the sternal third of clavicle; inequalities in the breath and stroke sounds; prolonged expiration; during full inspiration the air penetrates well the cells; vesicular murmur feeble, but distinctly audible; voice and cough sounds normal. Respiration much less rude on the left side than in July, scarcely indeed, more than puerile.

Case VI. I first saw in June, 1848, twelve months before the republication in this country of the paper of Dr. Williams. The patient was a locksmith, aet. 23 years, and had been the subject of phthisis for several years. The disease occupied a large portion of both lungs; had arrived at the period of ulceration with numerous anfractuosities in the clavicular and subclavicular regions; was accompanied by emaciation, cough, purulent expectoration, etc. etc. I prescribed the usual palliatives, gave the patient no hope or encouragement; expected him to die within a few months. In the autumn however, he traveled by slow stages, to New York, where he remained until October, 1849. While there he was directed to take cod-liver oil—commenced doing so in August. He said it was of great service to him, especially in arresting the hectic and night sweats, and alleviating the cough; his strength and appetite improved, and in October, he returned to Louisville. Called at my office for the purpose of having h s chest explored. I found that the deposition of tubercles had been apparently steady and uninterrupted—that anfractuosities existed where before there were none; in a word, that the disease at one or other of its stages involved almost the entire lung. Cough, hard, frequent; expectoration abundant, purulent; respiration short, accelerated; *no fever or night sweats;* appetite good, spirits cheerful; strength, he said, was improving. Advised to continue the oil. In two weeks after I was summoned in great haste to see the patient; found him laboring under intense pulmonary congestion of which he died in twenty-four hours. Autopsy was not made.

I have reported the above case simply for the purpose of illustrating the power of the oil in arresting the hectic fever and night sweats of phthisis. The disease of the lung was, I think, far too extensive even when I first saw the patient, to expect that the oil would produce any other than palliative effects. The patient, however, assured me that it had been of incalculable benefit to him.

Louisville, Ky., Feb. 27, 1851.

*Nashville Journal of Medicine and Surgery.*

